# A two-year field measurement of methane and nitrous oxide fluxes from rice paddies under contrasting climate conditions

**DOI:** 10.1038/srep28255

**Published:** 2016-06-20

**Authors:** Huifeng Sun, Sheng Zhou, Zishi Fu, Guifa Chen, Guoyan Zou, Xiangfu Song

**Affiliations:** 1 Eco-Environmental Protection Research Institute, Shanghai Academy of Agricultural Sciences, Shanghai 201403, China; 2 Shanghai Engineering Research Center of Low-carbon Agriculture (SERCLA), Shanghai 201415, China

## Abstract

The effects of three irrigation levels (traditional normal amount of irrigation [NA100%], 70%, and 30% of the normal amount [NA70% and NA30%]) and two rice varieties (*Oryza sativa* L. Huayou14 and Hanyou8) on CH_4_ and N_2_O emissions were investigated over two years under contrasting climate conditions (a ‘warm and dry’ season in 2013 and a normal season in 2014). Hanyou8 was developed as a drought-resistant variety. The mean seasonal air temperature in 2013 was 2.3 °C higher than in 2014, while the amount of precipitation from transplanting to the grain-filling stage in 2013 was only 36% of that in 2014. CH_4_ emission rose by 93–161%, but rice grain yield fell by 7–13% in 2013, compared to 2014 under the NA100% conditions. Surface standing water depths (SSWD) were higher in Hanyou8 than in Huayou14 due to the lower water demand by Hanyou8. A reduction in the amount of irrigation water applied can effectively reduce the CH_4_ emissions regardless of the rice variety and climate condition. However, less irrigation during the ‘warm and dry’ season greatly decreased Huayou14 grain yield, but had little impact on Hanyou8. In contrast, N_2_O emission depended more on fertilization and SSWD than on rice variety.

Modern changes in global climate, including greater atmospheric concentrations of greenhouse gases (CO_2_, CH_4_, N_2_O, etc.), temperature shifts, higher frequency of extreme events, such as drought or heavy rainfall, and heat waves, are directly or indirectly associated with the combustion of fossil fuels, land use change and other human activities that have occurred since the Industrial Revolution^1^. Such changes have greatly affected terrestrial ecosystems. For example, extreme events could lead to negative and even disastrous effects on plants^2^.

Rice (*Oryza sativa* L.) is the most widely consumed staple food crop in the world, and is particularly important in Asia. The food safety and national security of some countries depends on its production. Rice cultivation is also a significant source of greenhouse gas emissions, primarily methane (CH_4_) and nitrous oxide (N_2_O). Annual CH_4_ emissions from rice fields have been estimated to account for about 5–19% of global CH_4_ emissions, and agricultural N_2_O emissions increased by nearly 17% between 1990 and 2005, and now account for 60% of global anthropogenic N_2_O emissions^3^. The flooded environment, created during rice cultivation, provides anaerobic conditions that favor CH_4_ production by methanogens. The resulting CH_4_ can be oxidized by methanotrophs under aerobic conditions (e.g., in the rhizosphere and at the soil-water interface) and is finally emitted to the atmosphere through soil- or water-atmosphere interfaces and by the rice plant aerenchyma^4^. Nitrogen fertilization and water management (e.g. alternating wetting and drying) facilitates N_2_O emission via the processes of nitrification and/or denitrification in rice paddies^4^, hence, when applied appropriately, fertilizer and management interventions can play important roles in effectively controlling CH_4_ and N_2_O emissions during rice cultivation^5^,^6^,^7^,^8^,^9^. However, rice varieties differ significantly in terms of total CH_4_ and N_2_O emissions^10^,^11^,^12^,^13^. Selecting a rice variety that results in low CH_4_ and N_2_O emissions may therefore be a promising way to mitigate greenhouse gas emissions from rice paddies.

Drought is a serious limiting factor on crop production and the most damaging stressor in modern agriculture^14^,^15^. Rice consumes 70–90% of the total amount of irrigation water used in agriculture^16^,^17^, and as roughly a half of the world’s 158 million ha of rice land is paddy rice^18^, the production of this crop is very susceptible to water stress^19^. A series of agricultural practices, such as the use of saturated soil cultures and alternate wetting and drying, are recommended to reduce water input from irrigation and to enhance water use efficiency in rice cultivation. These practices could greatly reduce the water input required. However, they also lower rice grain yields to a certain extent^18^. Breeding new varieties of rice with drought tolerance may be an effective way of sustainably addressing the water scarcity issue^20^. These new varieties are called Water-saving and Drought-resistance Rice (WDR), and are characterized by having a similar yield potential and grain quality as wild type varieties, but require less water (50% less water use). In water-limited environments, they show higher drought resistance and minimize yield loss^20^. However, to our knowledge, little is known about the CH_4_ and N_2_O emissions from rice paddies by WDR under different climate conditions.

The annual mean surface air temperature for China over the past 97 years has experienced a warming of 0.79 °C, with a warming rate of 0.08 °C/10a, which is slightly larger than the global or northern hemispheric average as given by IPCC Third Assessment Report^21^. In addition, in the experimental region, the mean air temperature during the rice growing season in the 1990 s was 0.2 °C higher than that in the 1960–80s. However, the mean seasonal air temperature between 2001 and 2008 increased by 1.1 °C relative to that in the 1960–80s^22^. Climate models project that the increase in global surface air temperatures may exceed 1.5 °C by the end of the 21^st^ century relative to the average between 1850 and 1900^23^. Higher temperatures are known to influence water, ion, and organic solute movement across plant membranes, which interferes with photosynthesis and respiration. Excessive temperatures can reduce plant leaf photosynthesis and decrease the allocation of dry matter to the shoots and roots^24^. Therefore, high temperatures may have caused rice grain yield reductions in many rice growing areas^25^,^26^,^27^. Generally, weather patterns can be highly variable. For example, there may be little precipitation in one rice growing season and more in another. To satisfy the rice plant water demand, large amounts of water are used to irrigate rice fields in a low precipitation season, but less may be needed in a high precipitation season. Although some studies have demonstrated that CH_4_ and N_2_O emissions differed among rice growing seasons, little explanation has been provided for the variation^6^,^9^. Investigating the mechanisms underlying the differences in CH_4_ and N_2_O emissions under different climate conditions (air temperature and precipitation) is important when accurately assessing the total greenhouse gas emission caused by rice cultivation under global warming scenarios. Some process-based models (e.g. the Denitrification and Decomposition (DNDC) model) have been used to estimate the effects of changes in air temperature and precipitation on CH_4_ and N_2_O emissions from arable soils^28^,^29^. However, these results are not strongly supported by field experimental data. The field experiments examining the effects of climate change on CH_4_ and N_2_O emissions provide important information that may help improve long-term predictions with those models^30^.

In a previous study, we found that CH_4_ emissions fell significantly when less irrigation water was applied to rice paddies over two growing seasons^31^. In this study, the effects of two rice varieties (a WDR versus a common rice variety) and irrigation management technique on CH_4_ and N_2_O emissions from rice paddies were investigated over two growing seasons under contrasting climate conditions (a ‘warm and dry’ season and a normal season). In addition, the impacts of climate condition and irrigation management on rice grain yield and equivalent CO_2_ (CO_2_-eq) emission were also evaluated.

## Results and Discussion

### Irrigation management and SSWD dynamics under contrasting climate conditions

**Figure 1:**
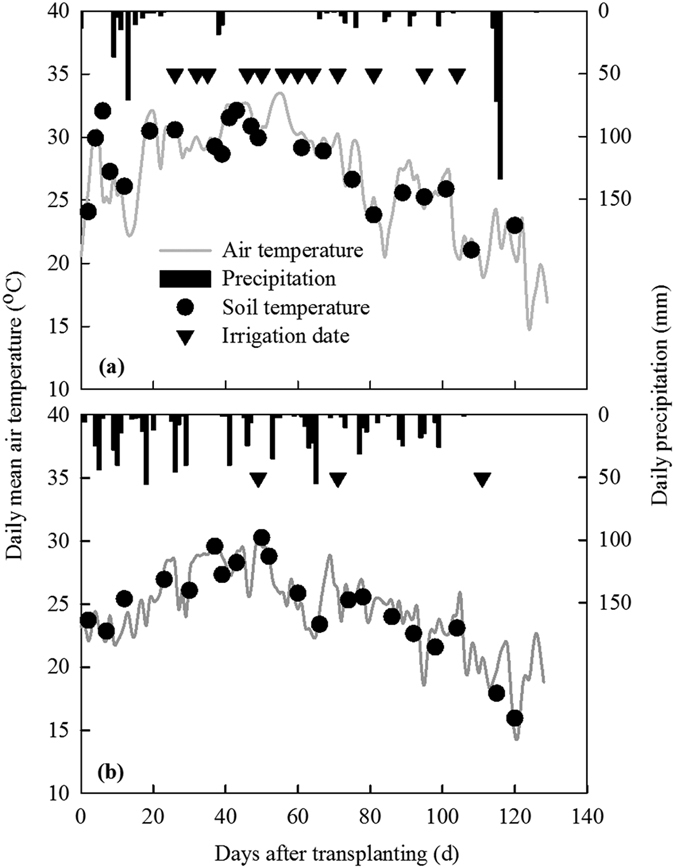
Seasonal changes in daily mean air temperature, daily precipitation, and soil temperature at 5 cm depth in the 2013 (**a**) and 2014 (**b**) rice growing seasons. Soil temperature values were obtained by averaging the values from all treatments during gas sampling events.

The total precipitation was 492.1 and 762.7 mm over the whole rice cultivation periods of 2013 and 2014, respectively (Table 1). In the ‘warm and dry’ rice season of 2013, only 271.8 mm (~55.2% of the total) of precipitation occurred from June to September, which is the period of active rice plant growth and development. In contrast, total precipitation in the normal rice season of 2014 occurred between June and September. Additionally, the total amount of evaporation was 591.6 and 481.6 mm in 2013 and 2014, respectively. Therefore, to satisfy the plant demand for water during growth and development, irrigation was applied up to 12 times in 2013 to keep the water table in the plots similar to traditional irrigation management (Fig. 1a). However, irrigation was applied only three times in 2014 (Fig. 1b). The total amount of irrigation in the NA100% plot was 611.7 mm in 2013 compared to only 116.7 mm in 2014 (Table 1). Consequently, the effective water inputs (precipitation + irrigation) from June to September for the NA100% plots were similar for the ‘warm and dry’ season (883.5 mm) and the normal season (879.4 mm). In contrast, the total water inputs in the NA70% and NA30% plots from June to September in 2013 were only 83% and 57% of those in 2014 (Table 1).

**Figure 2:**
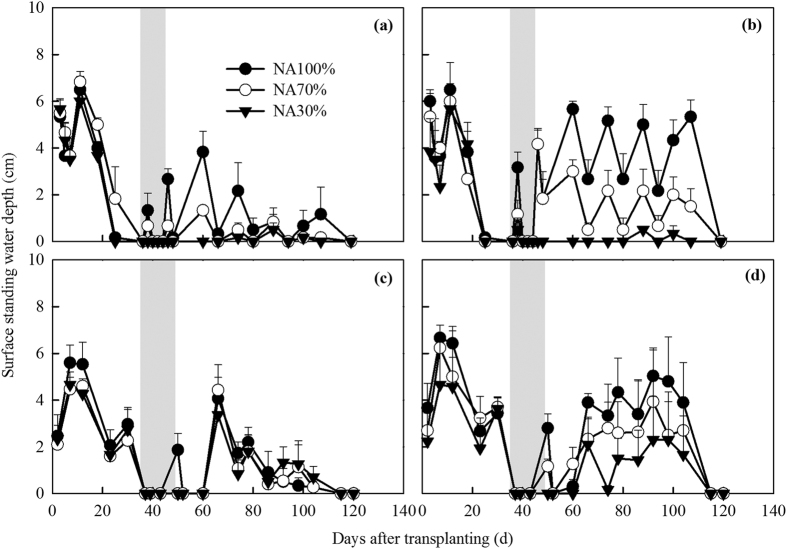
Seasonal variation in surface standing water depth (SSWD) in the 2013 (**a**) Huayou14; (**b**) Hanyou8 and 2014 (**c**) Huayou14; (**d**) Hanyou8 rice growing seasons. Abbreviations: NA100% = normal amount of irrigation, NA70 = 70% of normal irrigation, NA30 = 30% of normal irrigation. Gray belts represent the mid-season drainage periods. Error bars represent standard errors of the means.

Similar trends in SSWD were observed for all treatments in both seasons when the rice plants were small before MD (Fig. 2). The maximum SSWD reached similar values (approximately 6–7 cm). However, there were considerable differences in SSWD after MD between the rice varieties in 2013 (Fig. 2a,b). Under the same water inputs (precipitation + irrigation), the SSWDs of Hanyou8 in the NA100% and NA70% plots were significantly higher than those of Huayou14. Hanyou8 was developed as a drought-resistant variety and requires less water to maintain growth and development^20^. The average evapotranspiration rate for Hanyou8 was 5.4 mm day^−1^, a significantly lower rate than for Huayou14 (6.2 mm day^−1^), which probably resulted in higher SSWDs in the NA100% and NA70% plots when the WDR (Hanyou8) variety was grown compared to the common rice variety. However, the SSWD in the NA30% plots for both varieties was almost zero after MD in 2013 due to the reduced water input (Fig. 2a,b). In contrast, Hanyou8 mean SSWD was not significantly different to Huayou14 after MD in 2014. Furthermore, the SSWDs in the NA30% plots were similar to NA70% for both varieties in 2014 (Fig. 2c,d). Irrigation management appeared to have a lower effect on SSWD at the different irrigation levels due to higher precipitation in 2014.

### CH_4_ emissions under contrasting climate conditions

**Figure 3:**
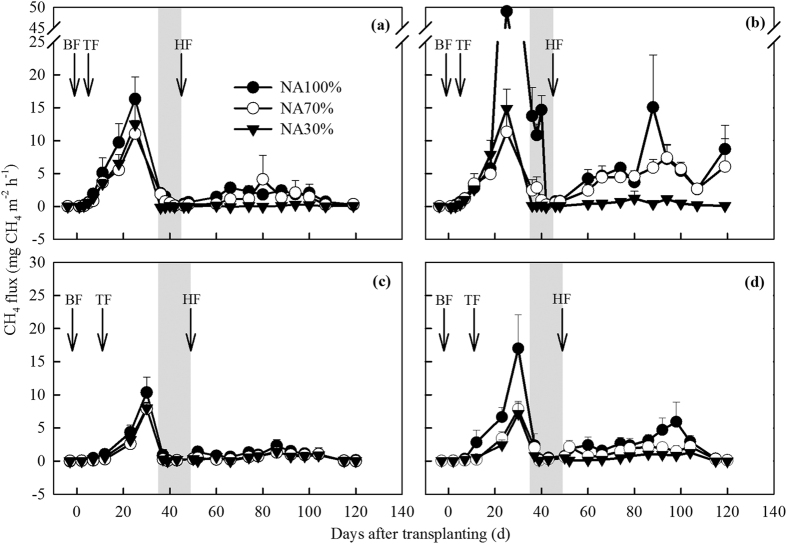
Seasonal variation in CH_4_ fluxes in the 2013 (**a**) Huayou14; (**b**) Hanyou8 and 2014 (**c**) Huayou14; (**d**) Hanyou8 rice growing seasons. Abbreviations: NA100% = normal amount of irrigation, NA70% = 70% of normal irrigation, NA30% = 30% of normal irrigation, BF = base fertilizer, TF = tillering fertilizer, HF = heading fertilizer. Gray belts represent the periods of mid-season drainage. Arrows denote the fertilization date, and error bars represent standard errors of the means.

The CH_4_ flux from all plots increased from the beginning, and peaked for the first time at 25–30 days after transplanting, then rapidly decreased to zero due to MD. Thereafter, CH_4_ fluxes from the NA100% and NA70% plots increased, peaked for a second time at 80–100 days after transplanting, and decreased afterwards until the end of the seasons. However, CH_4_ fluxes from the NA30% plot were very low after MD (Fig. 3). Consequently, the NA100% plots had the largest CH_4_ emissions for both rice varieties (Table 2). The average total CH_4_ emissions from the NA100% plots was 122 kg CH_4_ ha^−1^ (the range was from 47.8 to 252 kg CH_4_ ha^−1^ in this study), which was within the range of 5.4–275 kg CH_4_ ha^−1^ range recorded in numerous other studies where paddies were subject to traditional water management regimes (i.e. NA100% condition) and a N fertilizer application rate of 200–250 kg N ha^−1^, which is typical for the Yangtze River delta zone^7^,^9^,^32^,^33^,^34^,^35^.

A significant difference in total CH_4_ emissions was observed among irrigation levels (*P* < 0.01, Table 2). The NA70% and NA30% plots respectively reduced total CH_4_ emissions by 31–53% and 32–77% in both seasons compared to the NA100% plots. Similarly, Hou *et al.* reported that controlled irrigation (NA30%) mitigated CH_4_ emissions by 82% relative to traditional irrigation (NA100%)^33^. Wang *et al.* found that intermittent irrigation (corresponding to NA70%) and constant moisture (corresponding to NA30%) reduced CH_4_ emissions by 25% and 58%, respectively, compared to the continuous flooding condition (NA100%)^36^. Reductions in irrigation water to the rice paddies led to a lower SSWD and even no standing water above the surface. This increased oxygen penetration into the soil and led to soil organic C being oxidized to CO_2_ instead of CH_4_, which ultimately suppressed CH_4_ emissions. In this study, reducing the amount of irrigation was found to mitigate CH_4_ emissions not only under normal climate condition but also in the ‘warm and dry’ season.

There was a significant difference in CH_4_ emissions between Huayou14 and Hanyou8 (*P* < 0.05, Table 2). The CH_4_ emissions from Huayou14 were always lower than from Hanyou8, especially in the NA100% and NA70% plots. That may be related to the relatively lower SSWD in the Huayou14 plots compared to the Hanyou8 plots, which used less water to maintain growth and development and led to higher SSWDs (Fig. 2). The higher SSWD in the Hanyou8 plots could decrease soil Eh by 54 mV (averaged across the values for the NA100% and NA70% plots), and this facilitated CH_4_ production. Additionally, it has been well documented that the difference in CH_4_ emissions among rice varieties is correlated with the total amount of root exudate-C (e.g. sugars and organic acids)^37^,^38^. Similarly, in this study, some organic C materials, such as soluble sugar and proline, were observed higher in Hanyou8 than in Huayou14 (unpublished data), which may enhance CH_4_ production by changing the quantity and quality of the root exudates^39^. However, when the amount of irrigation water was reduced, CH_4_ emission was mitigated more in the Hanyou8 plots (average reduction of 52% and 73% by NA70% and NA30%, respectively, compared to NA100%) than that in the Huayou14 plots (average reduction of 34% and 42% by NA70% and NA30%,respectively, compared to NA100%) (Table 2).

CH_4_ emissions were significantly higher in the ‘warm and dry’ season of 2013 than in the normal season of 2014 (*P* < 0.01, Table 2). It is difficult to control climate conditions in field experiments, so there is little data available from field experiments but model simulations have been used to compare CH_4_ and N_2_O under contrasting climate conditions. By employing the DNDC biogeochemical model to simulate greenhouse gas emissions in Chinese rice growing fields between 1971 and 2010, Tian *et al.* found that CH_4_ emission was enhanced during the second 20 years (1991–2010) compared to the first 20 years (1971–1990) due to a 0.5 °C increase in air temperature^29^. In this study, the CH_4_ emissions from the NA100% plots by Huayou14 and Hanyou8 increased by 93% and 161% in the ‘warm and dry’ season of 2013, respectively, compared to the normal season of 2014. Our results partially confirmed the reliability of the model and they improved the accuracy and efficiency of the model when attempting to predict the effects of climate conditions on CH_4_ emissions in the future. There were several possible reasons for the relatively higher CH_4_ emissions in the ‘warm and dry’ season. Firstly, although there was less precipitation in 2013, large amounts of irrigation water were added to the plots to meet rice growth demand and made the SSWD values higher than or similar to those in 2014. Secondly, the higher air/soil temperature could potentially enhance CH_4_ production and emission^40^,^41^. Under higher air/soil temperature conditions, more root exudates are released into the soil and the potential availability of C for methanogens is enhanced^40^,^42^. For example, Tokida *et al.* found that soil warming enhanced rice root decay and provided more substrates for CH_4_ production^43^. Higher air temperatures also enhanced paddy soil organic C mineralization^44^ and potentially provided available C for methanogens. Moreover, the higher soil temperature around the rice roots may accelerate the CH_4_ transport process through rice plants^45^. Dijkstra *et al.* reviewed more than 100 rice paddy field studies that had investigated the response of CH_4_ and N_2_O emissions to elevated air/soil temperatures and reported that elevated temperature enhanced CH_4_ emissions in 73 studies, but depressed emissions in 41 studies^30^. These studies generally elevated the air/soil temperatures by using OTC (Open Top Chamber) or heating cables buried in the soil, or used infrared heaters installed above the canopy. However, they did not take the variations in precipitation into consideration. It is well known that the relative humidity of the atmosphere can have a negative relationship with the transpiration rate at certain temperatures. The lower relative humidity, caused by reduced precipitation and higher air temperatures in 2013, may have increased rice plant transpiration and allowed more CH_4_-rich water from underground to migrate aboveground, which would eventually increase the release of CH_4_ through micropores in the leaf sheaths.

### N_2_O emissions under contrasting climate conditions

**Figure 4:**
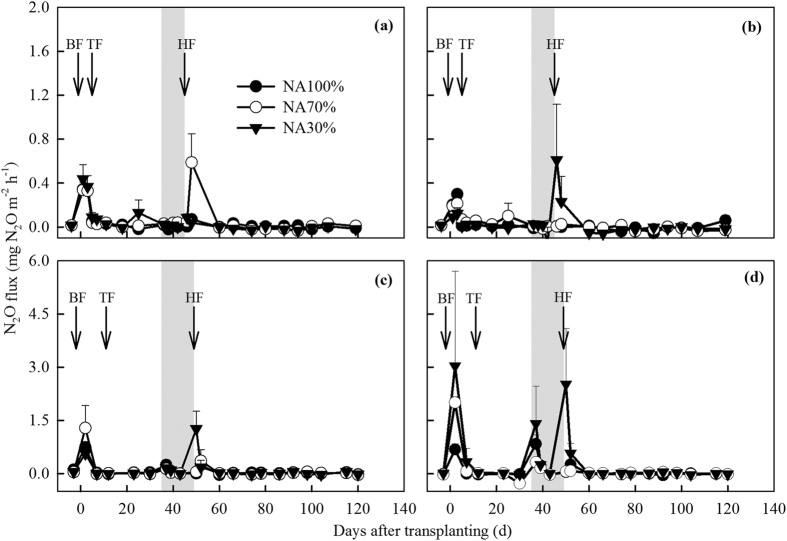
Seasonal variation in N_2_O fluxes in the 2013 (**a**) Huayou14; (**b**) Hanyou8 and 2014 (**c**) Huayou14; (**d**) Hanyou8 rice growing seasons. Abbreviations: NA100% = normal amount of irrigation, NA70 = 70% of normal irrigation, NA30 = 30% of normal irrigation, BF = base fertilizer, TF = tillering fertilizer, HF = heading fertilizer. Gray belts represent the periods of mid-season drainage. Arrows denote the fertilization date and error bars represent standard errors of the means.

As shown in Fig. 4, clear N_2_O fluxes peaks were detected mainly in the NA30% or NA70% plots for both varieties. However, at all the irrigation levels, no significant differences in N_2_O emissions were observed between Hanyou8 and Huayou14 in either season. Most of the N_2_O flux peaks were detected after fertilization, regardless of the rice variety. The influence of fertilization on N_2_O emissions was relatively transitory and vigorous (Fig. 4). The average total N_2_O emission was 1.2 kg N_2_O ha^−1^ and ranged from 0.2 to 2.0 kg N_2_O ha^−1^ in the NA100% plots, which was consistent with the values (0.4–5.3 kg N_2_O ha^−1^) recorded in many previous studies from the same region on paddies subjected to similar water management regimes and N fertilizer application rates^9^,^32^,^33^,^34^,^35^,^46^.

The NA70% and NA30% plots increased total N_2_O emissions by 22–146% and 26–338%, respectively, compared to the NA100% plots. However, no significant differences in total N_2_O emissions were detected amongst the irrigation treatments (Table 2). Hou *et al.* also found that N_2_O emissions under controlled irrigation (NA30%) increased by 135% relative to traditional irrigation (NA100%)^33^. Reductions in irrigation water to the rice paddies frequently subject the soil to alternating wet/dry conditions, which stimulates N_2_O producers activity and increases N_2_O emissions^4^. The increased N_2_O emissions in the NA70% and NA30% plots after fertilization, relative to the NA100% plot, are probably due to the abundant, newly-added N^6^ and the suitable soil moisture conditions^33^. Finally, there was no significant difference in total N_2_O emissions between Huayou14 and Hanyou8 (Table 2).

There were larger and more N_2_O flux peaks in the normal season of 2014 than in the ‘warm and dry’ season of 2013. Similarly, Tian *et al.* also suggested that an increase in precipitation could enhance N_2_O emissions after the DNDC model simulation^29^. Alternative wet and dry soil conditions caused by frequent precipitation would enhance N_2_O production through nitrification and denitrification in 2014^47^. However, in this study, the SSWD when fertilizer was applied may play an important role in controlling N_2_O emissions. The averaged SSWD over all treatments was higher after fertilization in the ‘warm and dry’ season of 2013 (5.3 cm and 1.9 cm after base and heading fertilization, respectively) than in the normal season of 2014 (2.6 cm and 1.0 cm after base and heading fertilization, respectively) (Fig. 2). In a freshwater marsh, Yang *et al.* also found that a lower water table position (−11 to 0 cm) enhanced N_2_O emissions relative to higher water tables (+2 to +14 cm)^48^. Similarly, in a northern boreal fen located in north-western Finland, Lohila *et al.* reported that the highest N_2_O fluxes occurred when the SSWD was about 4 cm, whereas atmospheric N_2_O was consumed when the SSWD was 15 cm^49^. When the SSWD is lower, less N_2_O dissolves into the surface standing water and more N_2_O is probably quickly released into the atmosphere. In contrast, higher SSWD could restrict the availability of oxygen and therefore favor the formation of molecular nitrogen (N_2_) instead of N_2_O. Hence, in this study, N_2_O emission might be more related to fertilization and SSWD rather than rice variety and high air temperature.

### Rice grain yield and equivalent CO_2_ emissions (CO_2_-eq) under contrasting climate conditions

In the NA100% plots, the average rice yield was 9.3 t ha^−1^ with a range of 8.7–10.1 t ha^−1^ (Table 3), which was higher than that recorded in other studies (4.8–9.3 t ha^−1^) conducted in the same region^7^,^9^,^32^,^33^,^34^,^35^,^46^. The potentially negative effect of less precipitation on rice grain yield may be offset by irrigation in the ‘warm and dry’ season. However, rice grain yield significantly decreased with the reduction in the amount of irrigation (*P* < 0.01, Table 3). In the ‘warm and dry’ season, the Huayou14 yield loss was 10% in the NA30% plots compared to the NA100% plots, which was greater than the yield loss recorded for Hanyou8 (6%). This indicates that Hanyou8 is more drought-resistant with regards to rice grain yield, which agrees with the results reported by Luo^20^.

Recently, Kim *et al.* used the CERES-Rice 4.0 crop simulation model to investigate the effects of climate change on rice grain yield in the temperate climate regions under the East Asian monsoon system and suggested that the air temperature increases could lead to a significant decrease in rice grain yield by 22.1–35.0%^50^. In this study, rice grain yield was significantly lower in the ‘warm and dry’ season (2013) than in the normal season (2014) (*P* < 0.001, Table 3). The yield decreased by 13–19% for Huayou14 and 7–12% for Hanyou8 in 2013 compared to the yield in 2014 and this was probably due to higher air temperature and reduced precipitation. In another case study conducted in the same region, Liu *et al.* reported that although the average rice grain yield increased by 46% from the 1980 s to 2000 s due to the soil improvement, rice variety updating and agricultural management advances, the climate conditions (e.g. higher air temperature, less precipitation) had a negative effect on rice grain yield^51^. Generally, air temperature enrichment in rice growing seasons could shorten rice development stages and reduce grain yield^52^,^53^,^54^. Elevated air temperature can result in rice grain yield loss, mainly through reduced photosynthesis caused by chloroplast damage, spikelet sterility caused by decreased pollen production, and increased energy consumption caused by higher respiration demand^25^. Moreover, there was a significant interaction between rice variety and year (*P* < 0.001, Table 3).

The average CO_2_-eq emissions in the NA100% plots was 378 kg CO_2_-eq t^−1^ with a range of 170 to 736 kg CO_2_-eq t^−1^ (Table 3), which was within the 33–557 kg CO_2_-eq t^−1^ range reported in previous studies conducted in the same region^9^,^32^,^33^,^34^,^35^. In most treatments, the detected CO_2_-eq emissions were higher in the ‘warm and dry’ season than in the normal season. However, the difference between the two seasons was not significant (Table 3). Higher CH_4_ emissions and lower rice grain yields from the treatments led to greater CO_2_-eq emissions in the ‘warm and dry’ season relative to the normal season. Interestingly, there was a significant difference in CO_2_-eq emissions between Huayou14 and Hanyou8 (*P* < 0.05, Table 3). This suggests that the CO_2_-eq emissions vary considerably with rice growing season or rice variety. Furthermore, CO_2_-eq emissions from the NA70% and NA30% plots of both rice varieties (excluding H8-NA30% in the 2014 normal season) were potentially depressed when compared to the NA100% plot (Table 3). The decreases in CH_4_ emissions from the NA70% and NA30% plots were the main cause of the effective depression in CO_2_-eq emissions, especially during the ‘warm and dry’ season. However, in the normal season, due to the higher N_2_O emissions, reductions in the amount of irrigation had little effect on CO_2_-eq emissions.

In conclusion, CH_4_ and N_2_O emissions strongly differed according to rice variety and irrigation management between the two rice growing seasons under contrasting climate conditions. The amount of irrigation water was significantly higher in the ‘warm and dry’ season than that in the normal season. Although the same amount of irrigation water was applied to the two rice varieties, the SSWDs in the plots planted with Hanyou8 were higher than Huayou14, due to lower water demand from Hanyou8. The CH_4_ emissions by Huayou14 and Hanyou8 increased 93% and 161% in the ‘warm and dry’ season (2013), respectively, compared with that in normal season (2014). Moreover, the CH_4_ emissions from Hanyou8 were higher than from Huayou14 in both seasons. Reducing the amount of irrigation water can effectively reduce the CH_4_ emissions, regardless of the rice variety and climate conditions. However, less irrigation during the ‘warm and dry’ season greatly decreased the Huayou14 grain yield, but had little impact on Hanyou8 yield. In contrast, compared to the effect of rice variety, N_2_O emission depended more on fertilization and surface standing water depth when the fertilizer was applied. Under the global warming scenarios, feasible reductions in the amount of irrigation water applied and the suitable selection of rice varieties would be a promising way to mitigate greenhouse gas emissions as well as maintain rice grain yield.

## Materials and Methods

### Study site and experimental design

The study was conducted in an experimental field at the Shanghai Engineering Research Center of Low-carbon Agriculture, which is a part of the Zhuanghang Experimental Station (30°53′N, 121°23′E), and is located in the Yangtze River delta zone in east China. A rice-wheat cropping rotation system is the typical practice in this area. The paddy field soil was plowed to a depth of ~15 cm, and its chemical and physical properties were as follows: soil organic C (SOC) 13.7 g·kg^−1^, total N 1.4 g·kg^−1^, bulk density 1.4 g·cm^−3^, and pH (H_2_O) 7.6.

Each experimental plot was 60 m^2^ and an impermeable membrane was buried vertically in the soil around each plot at 1.1 m depth to prevent lateral seepage between the experimental plots. Then a concrete wall (30 cm width × 60 cm height) was built around the experimental plots. It was half buried into the soil over the impermeable membrane. A centrifugal pump (SW100-160, 100 m^3^·h^−1^, Shanghai Sanxing Supply and Drainage Equipment Co., Ltd., Shanghai, China) and polyethylene pipes were used to transport river water to each plot for irrigation. A meteorological station was established nearby in 2012, which provided data about the air/soil temperature, dry/wet precipitation, evaporation, solar radiation, wind speed/direction etc.

Two rice varieties (*Oryza sativa* L. Huayou14 and Hanyou8) and three types of irrigation management were employed in this study. Each treatment was replicated three times, resulting in a total of 18 plots in this experiment (i.e., 2 rice varieties × 3 irrigation levels × 3 replicates). Huayou14 is a high-yielding hybrid that is often cultivated by local farmers and Hanyou8 was recently developed by the Shanghai Agrobiological Gene Center for its water-saving and drought-resistant traits. The three types of irrigation management applied were normal amount of traditional irrigation management (NA100%), 70% of normal (NA70%), and 30% of normal (NA30%). The performance of NA100% was consistent with conventional irrigation practice for meeting rice growth demand. The other two types were applied proportionately during every irrigation event when little or no surface-standing water was observed in the NA100% plot for Huayou14. The irrigation was mainly carried out between 30 and 100 days after transplanting.

### Climate conditions and agricultural practices

Seasonal changes in daily mean air temperature, daily precipitation, and soil temperature at 5 cm depth in the 2013 and 2014 rice growing seasons are shown in Fig. 1. The mean seasonal air temperature was 26.7 °C and ranged from 14.8 to 33.5 °C during the 2013 rice growing season (Table 1 and Fig. 1a), whereas it was 24.4 °C (ranging from 14.7 to 30.2 °C) in 2014 (Table 1 and Fig. 1b). The mean seasonal air temperature (24.4 °C) in 2014 was similar to the normal value (24.7 °C) reported by Su *et al.* in this region^55^. The mean seasonal air temperature in 2013 was 2.3 °C higher than that in 2014. Seasonal variations in soil temperature were similar to the daily mean air temperature (Fig. 1). Total precipitation in the 2013 and 2014 seasons was 492.1 and 762.7 mm, respectively (Table 1). In this region, annual precipitation is about 1200 mm, and about 60% of precipitation occurs between May and September^56^. In the 2013 season, precipitation between June and September (i.e., a duration from transplanting to the grain-filling stage) was only 271.8 mm, and this season was considered as a ‘warm and dry’ season. In contrast, in 2014, the total precipitation between June and September was similar to the average for the area, and this season was regarded as normal season.

Rice plants were transplanted at a density of 20 hills per m^2^ on June 14/16 and harvested on October 21/22 in 2013 and 2014, respectively. The N fertilizer application rate was 225 kg·ha^−1^, which was applied at a ratio of 5:3:2 (w/w/w) as base, tillering and heading applications, respectively. The base fertilizer was applied in the form of a compound fertilizer at 1–2 days before transplanting. The tillering and heading fertilizers were applied in the form of urea at about 1 week and 7 weeks after transplanting, respectively. Phosphorous (P_2_O_5_) fertilizer was applied as a base, compound fertilizer at a rate of 112.5 kg·ha^−1^, and 44% potassium (K_2_O) fertilizer was applied as a base, compound fertilizer at a rate of 255 kg·ha^−1^. The remaining of potassium (K_2_O) fertilizer was applied as a heading fertilizer in the form of commercial potassium chloride (KCl).

There were 12 and three irrigation events in the 2013 and 2014 seasons, respectively. Mid-season drainage (MD) is a conventional agricultural practice during the rice growing season. The MD began on July 19/21 and finished on July 29/August 4 in 2013 and 2014, respectively.

### Measurements

The samples used to determine CH_4_ and N_2_O concentrations were taken using a static transparent chamber consisting of a plexiglass base frame (50 cm length × 40 cm width × 20 cm height) and a plexiglass lid (50 cm × 40 cm × 50 cm) equipped with a battery-driven 12 V fan at the center of its inner top. Other plexiglass frames were used to extend the lid height, by 20, 40, or 60 cm depending on the height of rice plants. The base frames were inserted approximately 15 cm into the soil, and four hills of rice plants were transplanted. One base frame was placed in each plot. Four gas samples were collected from each chamber at 6-min intervals using an auto gas sampler attached to four aluminum foil gas bags (1 L, Dalian Delin Gas Packing Co., Ltd., Dalian, China) at each sampling time. The auto gas sampler was composed of a 12 V rechargeable battery (NP7-12, YUASA Battery (Guangdong) Co., Ltd, Guangzhou, China), a gas pump (FAY4002, 2 L min^−1^, Chengdu Qihai E&M Manufacturing Co., Ltd., Chengdu, China), a box containing a circuit board (Nanjing Weina Electronic Co., Ltd., Nanjing, China), and a series of compact direct-operated 2-port solenoid valves (VDW23-6 G-1, SMC Pneumatics Ltd., Tokyo, Japan).

The gas samples from all the plots were collected between 08:00 and 10:00 and immediately taken to the laboratory. The concentrations of CH_4_ and N_2_O were determined by a gas chromatograph (7820 A, Agilent Technologies, Inc., Santa Clara, CA, USA) equipped with a flame ionization detector and an electron capture detector, respectively. The sampling frequency was once a week. However, whenever there was a fertilizer application, an MD, or irrigation after MD, a higher sampling frequency (once every 2 days) was used and daily sampling lasted for one week. CH_4_ and N_2_O flux were calculated by examining the linear increases of CH_4_ and N_2_O concentrations in the headspace of the chambers over time. The seasonal total CH_4_ and N_2_O emissions from all plots were calculated directly from the fluxes.

The surface-standing water depth (SSWD) was measured directly using a ruler after each gas-sampling event. Soil Eh (Oxidation-Reduction Potential) was detected at 5 cm soil depth with a pH/NO_3_/Eh meter (PRN-41, DKK-TOA Co., Tokyo, Japan). Soil temperature was monitored at 5 cm soil depth using a moisture meter (HH2, Delta-T Devices Ltd, Cambridge, UK) during each gas-sampling event around the base frames. At the end of each rice growing season, the plants in each plot were manually harvested. The dry weight of the rice grains was determined using an oven at 75 °C. Finally, the rice grain yield of each plot was calculated on a rice grain dry weight basis using the equation:


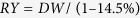


where *RY* is the rice grain yield (t·ha^−1^), *DW* is the dry weight of rice grains (t·ha^−1^), and 14.5% was used as the standard moisture content for storage of the rice varieties used in this study.

The equivalent CO_2_ (CO_2_-eq) emission for total CH_4_ and N_2_O emissions (greenhouse gas intensity) was calculated on a rice grain yield basis using the equation:


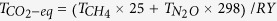


where *T*_CO2-eq_ is the total amount of equivalent CO_2_ emission (kg CO_2_-eq·t^−1^), *T*_CH4_ is the total amount of CH_4_ emission (kg·ha^−1^), *T*_N2O_ is the total amount of N_2_O emission (kg·ha^−1^), 25 and 298 are the multiples of GWP (global warming potential) for CH_4_ and N_2_O versus CO_2_ over 100 years^3^, and *RY* is the rice grain yield (t·ha^−1^).

### Statistical analysis

The impacts of the three parameters (irrigation management, rice variety, and year) on CH_4_ and N_2_O emissions from rice paddies were examined. Their effects were analyzed using the general linear model for analysis of variance along with the least significant difference test. The significance level for both tests was 5%. SPSS 20.0 statistical software (IBM Co., New York, USA) was used to conduct the analysis. The figures were prepared using Sigmaplot 12.5 software (Systat Software Inc., San Jose, CA, USA).

## Additional Information

**How to cite this article**: Sun, H. *et al.* A two-year field measurement of methane and nitrous oxide fluxes from rice paddies under contrasting climate conditions. *Sci. Rep.* **6**, 28255; doi: 10.1038/srep28255 (2016).

## Figures and Tables

**Table 1:** Mean seasonal air and soil temperatures, total precipitation, and amount of irrigation water in the 2013 and 2014 rice growing seasons.

Rice season	Treatments	Mean seasonal air temperature (°C)	Mean seasonal soil temperature (°C)	Seasonal total precipitation (mm)	Precipitation from June to September (mm)	Amount of irrigation (mm)	Water input from June to September (mm)
2013 (‘warm and dry’)	NA100%	26.7	27.8	492.1	271.8	611.7	883.5
	NA70%	26.7	27.7	492.1	271.8	428.3	700.1
	NA30%	26.7	28.0	492.1	271.8	185.0	456.8
2014 (normal)	NA100%	24.4	24.7	762.7	762.7	116.7	879.4
	NA70%	24.4	24.7	762.7	762.7	81.7	844.4
	NA30%	24.4	24.8	762.7	762.7	35.0	797.7

NA100% = normal amount of irrigation, NA70% = 70% of normal irrigation, NA30% = 30% of normal irrigation.

**Table 2:** Seasonal total CH_4_ and N_2_O emissions in the 2013 and 2014 rice growing seasons.

Treatments	CH_4_ emission (kg CH_4_ ha^−1^)		N_2_O emission (kg N_2_O ha^−1^)	
	2013	2014	2013	2014
H14-NA100%	92.3 ± 18.8	47.8 ± 14.7	0.6 ± 0.2	1.8 ± 0.4
H14-NA70%	63.4 ± 19.9	30.6 ± 6.0	1.5 ± 0.5	2.2 ± 0.8
H14-NA30%	44.9 ± 3.2	32.6 ± 4.9	0.8 ± 0.4	2.7 ± 0.6
H8-NA100%	252.2 ± 108.2	96.5 ± 31.4	0.2 ± 0.3	2.0 ± 0.8
H8-NA70%	117.8 ± 17.8	47.9 ± 16.7	0.4 ± 0.3	2.6 ± 0.2
H8-NA30%	58.7 ± 13.3	29.7 ± 6.2	0.5 ± 1.0	8.9 ± 6.4
Analysis of variance				
IM	**		*ns*	
RV	*		*ns*	
Year	**		*	
IM × RV	*ns*		*ns*	
IM × Year	*ns*		*ns*	
RV × Year	*ns*		*ns*	
IM × RV × Year	*ns*		*ns*	

Numbers in the table represent means ± standard errors. H8 = Hanyou8, H14 = Huayou14, NA100% = normal amount of irrigation, NA70% = 70% of normal irrigation, NA30% = 30% of normal irrigation, IM = Irrigation management, RV = Rice Variety. * *P* < 0.05, ** *P* < 0.01, ns = not significance at 0.05 level.

**Table 3:** Rice grain yield and CO_2_-eq emissions in the 2013 and 2014 rice growing seasons.

Treatments	Rice grain yield (t ha^−1^)		CO_2_-eq emission (kg CO_2_-eq t^−1^)	
	2013	2014	2013	2014
H14-NA100%	8.8 ± 0.1	10.1 ± 0.2	283.1 ± 48.2	169.7 ± 25.2
H14-NA70%	8.3 ± 0.2	10.0 ± 0.2	246.4 ± 73.9	142.2 ± 39.2
H14-NA30%	7.9 ± 0.0	9.8 ± 0.1	170.2 ± 9.0	165.8 ± 25.8
H8-NA100%	8.7 ± 0.1	9.4 ± 0.1	735.7 ± 324.8	322.8 ± 69.3
H8-NA70%	8.6 ± 0.3	9.3 ± 0.1	352.9 ± 30.7	211.5 ± 45.8
H8-NA30%	8.2 ± 0.4	9.3 ± 0.1	194.1 ± 58.1	371.9 ± 201.7
Analysis of variance				
IM	**		*ns*	
RV	*		*	
Year	***		*ns*	
IM × RV	*ns*		*ns*	
IM × Year	*ns*		*ns*	
RV × Year	***		*ns*	
IM × RV × Year	*ns*		*ns*	

Numbers in the table represent means ± standard errors. H8 = Hanyou8, H14 = Huayou14, NA100% = normal amount of irrigation, NA70% = 70% of normal irrigation, NA30% = 30% of normal irrigation, IM = Irrigation management, RV = Rice Variety. *P < 0.05, **P < 0.01, ***P < 0.001, ns = not significance at 0.05 level.
